# Complex Interaction of Sensory and Motor Signs and Symptoms in Chronic CRPS

**DOI:** 10.1371/journal.pone.0018775

**Published:** 2011-04-29

**Authors:** Volker Huge, Meike Lauchart, Walter Magerl, Antje Beyer, Patrick Moehnle, Wibke Kaufhold, Gustav Schelling, Shahnaz Christina Azad

**Affiliations:** 1 Department of Anaesthesiology, Ludwig-Maximilians-Universität München, Munich, Germany; 2 Center of Biomedicine and Medical Technology (CBTM), Department of Neurobiology, Medical Faculty Mannheim, Ruprecht-Karls-Universität Heidelberg, Mannheim, Germany; Tokyo Institute of Psychiatry, Japan

## Abstract

Spontaneous pain, hyperalgesia as well as sensory abnormalities, autonomic, trophic, and motor disturbances are key features of Complex Regional Pain Syndrome (CRPS). This study was conceived to comprehensively characterize the interaction of these symptoms in 118 patients with chronic upper limb CRPS (duration of disease: 43±23 months). Disease-related stress, depression, and the degree of accompanying motor disability were likewise assessed. Stress and depression were measured by Posttraumatic Stress Symptoms Score and Center for Epidemiological Studies Depression Test. Motor disability of the affected hand was determined by Sequential Occupational Dexterity Assessment and Michigan Hand Questionnaire. Sensory changes were assessed by Quantitative Sensory Testing according to the standards of the German Research Network on Neuropathic Pain. Almost two-thirds of all patients exhibited spontaneous pain at rest. Hand force as well as hand motor function were found to be substantially impaired. Results of Quantitative Sensory Testing revealed a distinct pattern of generalized bilateral sensory loss and hyperalgesia, most prominently to blunt pressure. Patients reported substantial motor complaints confirmed by the objective motor disability testings. Interestingly, patients displayed clinically relevant levels of stress and depression. We conclude that chronic CRPS is characterized by a combination of ongoing pain, pain-related disability, stress and depression, potentially triggered by peripheral nerve/tissue damage and ensuing sensory loss. In order to consolidate the different dimensions of disturbances in chronic CRPS, we developed a model based on interaction analysis suggesting a complex hierarchical interaction of peripheral (injury/sensory loss) and central factors (pain/disability/stress/depression) predicting motor dysfunction and hyperalgesia.

## Introduction

Complex Regional Pain Syndrome (CRPS), mostly regarded as a neuropathic pain disorder, is typically evolving after a minor trauma of the limb [1]. Besides pain, CRPS displays a multifaceted clinical pattern consisting of vaso- and sudomotor changes, as well as trophic and motor disturbances, edema and somatosensory changes [2]. In consequence, many patients sustain impairments of hand function persisting even many years after the initial trauma [3]. The clinical presentation, and therefore the criteria leading to the diagnosis of CRPS, are mostly applied to patients with recently emerging, “acute” CRPS [1], [4]. Much less is known about the occurrence of the respective signs and symptoms when the initial phase of the disease subsides. Furthermore, the underlying pathophysiology of CRPS is still under debate [5]. Some authors stress the role of peripheral pathomechanisms, namely peripheral neurogenic inflammation and small fiber axonal degeneration [6], [7]. In addition, autoimmune dysfunction seems to be involved in CRPS pathomechanisms [8]. Contrariwise, a distinguished body of literature supports the involvement of the central nervous system in terms of sensory as well as motor adaptive changes [9], [10]. More generally, the level of accompanying chronic stress and depression might also account for somatosensory changes and the level of ongoing or evoked pain particularly in chronic pain patients [11], [12]. However, the degree of stress and depression in patients with chronic CRPS is not well characterized. Recently, it has been suggested that the pathophysiological mechanisms of CRPS follow a distinct time course, with a preponderance of peripheral inflammation and beginning of small fiber degeneration in the acute phase, and progression of small fiber degeneration as well as central pathomechanisms dominating the chronic phase of the disease [13]. It is still unclear to which degree the underlying pathophysiological mechanisms predict the clinical presentation of CRPS and the resulting outcome of the disease, although recent studies suggest an interdependency between the clinical presentation, the underlying pathophysiology and possible consequences in terms of resulting impairments. Namely, differences in skin temperature might facilitate the discrimination between an ongoing peripheral or central pathophysiology. [14]. So far, many clinical studies focused on the characterization of different specific aspects of the disease, for example the degree of neurological changes or the description of motor impairments [15], [16]. Furthermore, many studies mixed patients with short duration of the disease with those suffering from chronic CRPS. Up to now, a comprehensive survey linking quantitative sensory changes to CRPS symptomatology and the degree of resulting impairment is still unavailable for patients with chronic CRPS. In order to expand the knowledge of clinical characteristics of chronic CRPS and the level of concomitant stress and depression, as well as to characterize the degree of resulting hand impairment and disability, this study was performed.

## Material and Methods

### Patients and Treatment

**Figure 1:**
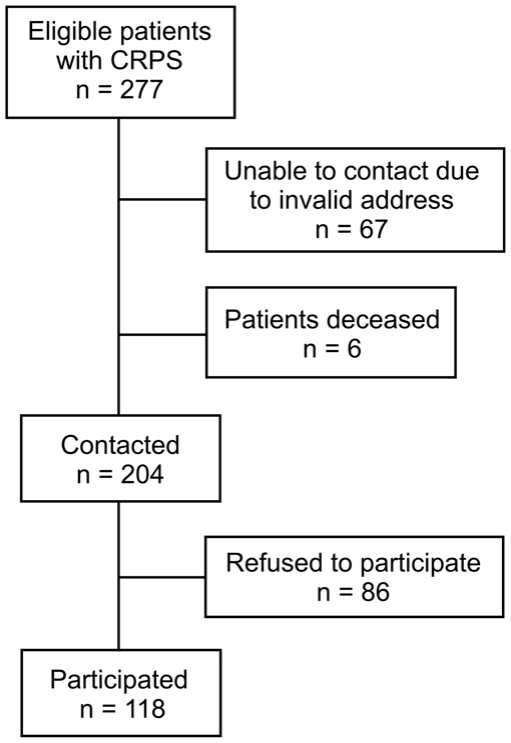
Patient disposition. Disposition of patients eligible for the study. Eligible patients had been treated at the pain clinic and were diagnosed with chronic CRPS (Duration of disease more than 12 months).

All patients with a history of CRPS of more than 12 months diagnosed either by the IASP criteria or the research diagnosis criteria proposed by Bruehl (Table 1) [2], who had been treated at the pain clinic of the University of Munich, were contacted by mail and asked to participate in the study. Until 2001 clinical diagnosis of CRPS was established by using the IASP criteria. From 2001, CRPS was diagnosed at our pain clinic by using the revised diagnosis criteria of Bruehl (Table 1). A total number of 277 patients were identified using computerized data processing, of which 118 patients gave written informed consent and participated in the study. The remaining 159 patients could not be contacted due to an invalid address or refused to participate in the study. The exact patients' disposition is illustrated in *Figure 1*. The study was approved by the local ethics committee (Ludwig Maximilian University of Munich, ethics committee), and written informed consent was obtained by all subjects enrolled in the study according to the Declaration of Helsinki. All patients were insured against travel accidents (Insurance Policy Number: 08.715506462). Furthermore, all patients received an allowance of 20 € for participation in the study.

**Table 1:** Proposed modified research diagnostic criteria for CRPS.*

***1: Continuing pain which is disproportionate to any inciting event***	
***2: Must report at least one symptom in each of the four following categories***• **Sensory:**reports of hyperesthesia	***3: Must display at least one sign in two or more of the following categories***• **Sensory:**evidence of hyperalgesia (to pinprick) and/or allodynia (to light touch)
• **Vasomotor:**reports of temperature asymmetry and/or skin color changes and/or skin color asymmetry	• **Vasomotor:**evidence of temperature asymmetry and/or skin color changes and/or asymmetry
• **Sudomotor/edema:**reports of edema and/or sweating changes and/or sweating asymmetry	• **Sudomotor/edema:**evidence of edema and/or sweating changes and/or sweating asymmetry
• **Motor/trophic:**reports of decreased range of motion and/or motor dysfunction (weakness, tremor, dystonia) and or throphic changes (hair, nail, skin)	• **Motor/trophic:**evidence of decreased range of motion and/or motor dysfunction (weakness, tremor, dystonia, and/or trophy changes (hair, nail, skin)

*Bruehl S, Harden RN, Galer BS et al. External validation of IASP diagnostic criteria for Complex Regional Pain Syndrome and proposed research diagnostic criteria. International Association for the Study of Pain. Pain 1999; 81: 147–154.

Patients suffering from CRPS had been treated following the treatment guidelines of our pain clinic: Treatment invariably contained physical therapy, occupational therapy and lymph drainage as long as clinical signs of edema were present. Drug therapy contained a WHO ladder step I medication (preferably nonsteroidal antiphlogistics), as well as a drug recommended as a first line choice for the treatment of neuropathic pain conditions [17] (Amitriptyline or Gabapentin). In cases of insufficient pain control (NRS>4), a WHO ladder step II opioid medication (Tramadol or Tilidine/Naloxone) had been given in addition. Choice of drug as well as drug dosing had been left to the discretion of the attending physician. In case of consent, patients had undergone stellate-ganglion block in order to test for possible sympathetically maintained pain (SMP).

### Quantitative Sensory Testing

All patients received a standardized Quantitative Sensory Testing (QST) for characterization of a complete somatosensory phenotype. Testing contained establishment of sensory as well as thermal detection and pain thresholds, vibration thresholds, mechanical pain sensitivity, paradoxical heat sensation and pressure pain thresholds. All tests were performed under minimal distraction in a silent, air-conditioned room, with an ambient temperature of 25–26°C. Subjects were seated on a comfortable chair, and allowed to adapt to the test environment for at least 20 minutes. The course of assessments was explained to the subjects by written standard patient instructions. QST followed the protocol suggested by the German Research Network on Neuropathic Pain (DFNS). All sensory tests were demonstrated in a remote test area (forearm) not affected by the underlying disease. The hand affected by the disease was termed “ipsilateral”, while the other hand was termed “contralateral”. All tests were performed at the dorsum of the ipsi- as well as the contralateral hand, starting at the contralateral side. Thermal testing was performed using a Medoc Thermal Stimulus Analyser TSA-2001 device (Medoc, Ramat Yishai, Israel) with a computer-controlled Peltier-based probe. Thermal testing consisted of testing for Cold Detection Threshold (CDT), Warm Detection Threshold (WDT), Cold Pain Threshold (CPT) and Heat Pain Threshold (HPT) and Thermal Sensory Limen (TSL) by using the methods of limits. Furthermore, a testing for elicitation of Paradoxical Heat Sensations (PHS) was applied. Mechanical testing consisted of determination of Mechanical Detection Threshold (MDT), Vibration Detection Threshold (VDT), Mechanical Pain Threshold (MPT), Mechanical Pain Sensitivity (MPS), Wind-up Ratio (WUR) and Pressure Pain Threshold (PPT). Furthermore, the degree of Dynamic Mechanical Allodynia (DMA) was assessed. Total duration of sensory testing was about 60 minutes. For an elaborate discussion see Rolke et al. [18], [19].

### Clinical Assessment

Infrared thermometry (Proscan 510; Dostmann Electronic, Wertheim-Reicholzheim, Germany) was used for clinical assessment of sympathetic outflow [20]. Skin temperature was measured three times on glabrous skin aside from skin veins on the back of the hand, followed by three measurements on the palm of the hand. Emissivity was set to 0.96 (i.e. near black body emissivity). The arithmetic mean was used for further data processing. Mean temperature differences between the ipsilateral and the contralateral skin temperature were calculated in order to discriminate patients with “warm” from those with “cold” CRPS. A difference of ≥1°C was assumed to be clinically relevant.

Hand edema was assessed using a custom made Lucite volumeter (13.5*13.5*34.5 cm), with the patient in an upright position. The testing commenced as described by Stern [21], who established reference values with the mean volumes of dominant hands about 9 ml bigger than those of non-dominant hands (test-retest reliability r = 0.91–0.99) [21]. Side-to-side differences exceeding 5% were considered as pathologic in patients suffering from CRPS [22].

A standardized clinical examination of CRPS symptomatology according to the IASP criteria was performed in all patients. The testing commenced in a given sequence in a silent air-conditioned room at 23–25°C. Patients were allowed to adopt the environmental terms for at least 15 minutes before testing. All clinical examinations were performed by one of three experienced examiners (VH; AB; WK). Assessment started with clinical testing for mechanical hyperalgesia, mechanic dynamic allodynia as well as hypoaesthesia. Mechanical hyperalgesia was tested using a blunt copper wire with a diameter of 1 mm. Mechanic dynamic allodynia and hypoaesthesia were tested using a mounted Q-tip. All examinations were carried out on the dorsum of the ipsi- as well as the contralateral hand, beginning with the contralateral side. Mechanical hyperalgesia was accounted when painful sensation elicited on one examined area was considerably more pronounced than on the contralateral side.

Mechanical allodynia was considered to be present if pain was evoked by slightly touching the examined areal. Mechanical hypoaesthesia (loss of touch sensation) was diagnosed when touch sensation on one side was diminished in side to side comparison. Sudomotor dysfunction was evaluated in a dichotomous way (Sweating abnormality: present/absent) in comparison to the contralateral side. Disturbances of hair as well as nail growth, changes in skin colour, presence of edema and skin gloss were judged in an identical manner.

An 11 point Likert scale ranging from 0–10 (Numeric rating scale NRS) was used to assess the patients' subjective intensity of spontaneous ongoing pain. Furthermore, evoked pain accompanying physical strain of the hand was evaluated in an assessed as either exaggerated by physical strain or not (i.e. present or absent). Moreover, presence or absence of shoulder or elbow pain was recorded.

The assessment of hand motion took place in an analogous setting. Patients underwent goniometric measurements to determine active range of motion (AROM) of the wrist (extension/flexion) as well as thumb abduction by using a standard plastic transparent goniometer. AROM was defined as the maximum amount of joint motion attained by a subject during active performance of joint motion. Patients were instructed to move the respective joint as far as possible. For wrist extension and flexion a test-retest reliability of >0.90 and inter-observer reliability coefficients between 0.78 and 0.91 have been reported [23]. Furthermore, the diameter of the hand between D1 and D5 during maximal active finger extension was quantified in cm (diameter D1–D5), and failure of maximal finger extension in contrast to the not affected hand was registered (maximal Finger extension possible/not possible). Moreover, patients were requested to actively flex the distal as well as the proximal interphalangeal joints (so called “clenching a small fist”). Afterwards, patients were asked to flex the metocarpophalangeal joints as far as possible (so called “clenching a big fist”). An insufficiency to perform one of the tasks was recorded (possible/impossible). Furthermore, the maximal deflection deficit between the most affected finger and the hands' palm was quantified (Deflection Deficit). Patients were asked to oppose D1 and D5. Any deficits in opposition were quantified (Opposition Deficit D1–D5). All measurements were carried out on both hands respectively. The patients' ability to supinate or pronate the wrist as well as the forearm was evaluated and incapacities were recorded (Supination/Pronation: possible; partially possible; impossible). Finally, the patients' ability for abduction and external rotation of the shoulders was tested. The patients were asked to place their hands behind their head and were instructed to reach as far down their spine as possible. Ability for adduction combined with internal rotation of the shoulders was evaluated by asking the patients to place their hands behind their back and to reach as high up their spine as possible. Failure to join hands was recorded as pathological outcome of the tests.

The muscle strength was measured by means of a hand held dynamometer (*CITEC: Center of Innovative Technic BV; Netherlands*) following a standardized protocol. The utilized dynamometer measures grip strength in Newton (N) with an accuracy of 0.1%. All measurements were performed on the ipsi- as well as the contralateral hand. During the tests, the display was invisible for the patient. Each test was performed three times each hand, and the respective arithmetic mean was used for further analysis. Testing commenced with the patient in a seated position in the environment described above. Patients were instructed to apply the maximal possible force. Full-fist grip, three point grip and pinch-grip were measured [24]. Full fist grip: The patient clinched his fist around the applicator. Three point grip: Distal phalanx of the thumb was positioned under the device, whereas the distal two phalanges of the 2^nd^ and 3^rd^ fingers were positioned above it. Pinch Grip: The distal phalanx of the thumb was placed above the device, while the radial side of the index fingers' middle phalanx was positioned under it.

Hand-related dexterity was evaluated via the “Sequentional Occupational Dexterity Assessment” (SODA) [25]. The test measures hand dexterity, defined as a complex of bimanual functional abilities in activities of daily living and was especially designed for patients with rheumatoid arthritis. Tasks of the SODA inter alia contain picking up an envelope, or unscrewing the cap of a tube of toothpaste. Assessment took place under the same conditions as described above. Six tasks of the SODA are unilateral, and six are bilateral. In the unilateral tasks the patient was requested to use the less affected hand. The investigator rated the subject performance on each item (0 = unable to perform the task; 1 = able to perform the task in a different way, 4 = able to perform the task as requested). Furthermore, after each task the patient was asked whether the task was difficult to perform (0 = very difficult; 1 = some difficulty, 2 = not difficult). Summation of both scores resulted in an evaluation score for each task from 0–6 (0 = unable to perform the task; 6 = able to perform the task as requested without any difficulty). Scores on the individual tasks were summed, resulting in a total SODA score from 0–108. Furthermore, patients were asked whether performing the tasks of the SODA was painful or not, and the SODA-pain score was determined (range 0–12). For a more detailed discussion of the test see [25]. To determine hand-specific disability, the Michigan Hand Outcomes (MHQ), a health and functional status questionnaire designed specifically for assessment of the hand, was used. The questionnaire contains six distinct scales: (1) overall hand function, (2) activities of daily living, (3) pain, (4) work performance, (5) aesthetics, (6) patient's satisfaction with hand function. Scoring and interpretation of the data followed an algorithm described by Chung and colleagues [26].

### Psychological Assessment

The German version of the Pain Disability Index (PDI) was used to measure the pain-related interference with seven distinct domains of daily life [27].

Symptoms of depression were assessed by means of the German version of the Center for Epidemiological Studies Depression Test (CES-D). This test combines twenty questions designed to measure levels of depression [28], [29]. A raw test score of 27 or more is considered to be the critical limit for the presence of a depressive episode in pain patients [30].

Intensity and incidence of stress symptoms were measured using the German Version of the Post-Traumatic Stress Symptom 10-Questionnaire (PTSS-10) [31]. The PTSS-10 detects the presence and intensity of stress symptoms as for example sleep disturbances, nightmares and generalized irritability. The symptoms are rated by the patients in a scale from 1 (never) to 7 (always). A summary score >35 is associated with a high probability of fulfilling the diagnostic criteria for posttraumatic stress disorder (PTSD). Moreover, all patients completed a validated questionnaire evaluating different categories of traumatic memory occurring in the week before the assessment. The number of traumatic memories recalled was indexed [32].

The German version of the Short Form 36 (SF-36) was employed to assess the overall health-related quality of life. The SF-36 is a generic measure independent of age and underlying disease, assessing the generic health-related quality of life (HRQL) in eight dimensions (physical functioning, role physical, bodily pain, general health, vitality, social functioning, role emotional, mental health) aggregated into two summary scores (physical and mental health; PCS/MCS). The eight scales and the two summary scores are scored from 0 to 100, with 0 indicating the worst health and 100 the best [33].

### Data analysis

Nonparametric paired Wilcoxon signed ranks method test was applied to detect side-to-side differences for scores with nominal or ordinal data level. Paired t-test was used for side-to-side comparisons as well as for group comparisons for data on interval level.

All QST data except CPT/HPT/VDT and PHS were transformed into decadic logarithms to achieve secondary normal distributions [18]. To be able to compare sensory data across different QST parameters data were further transformed into standard normal distributions (z-normalized) relative to reference data of the DFNS cohort of healthy subjects [19]. Briefly, all patient data were normalized to the respective gender and age group of healthy controls using the equation: z = (individual value−mean_reference data base_)/SD_reference data base_. Significance of differences from healthy controls was estimated comparing the patients mean ± SD obtained by this z-normalization vs. a standard normal distribution (i.e. mean ± SD = 0±1) of an equal number of healthy subjects of the DFNS reference data using the web-based statistical freeware (Simple Interactive Statistical Analysis SISA, Uitenbroek 1997; http://home.clara.net/sisa/binomial.htm) [34]. Recently, QST has been demonstrated to show a high test-retest and inter-observer reliability, thereby enabling a comparison of QST results with the age and gender matched healthy control of the DFNS [35]. Data of SF36 were normalized as described above to a representative sample of the US-General population (n = 2393).

In order to explore functional interdependence between pain, sensory, motor and psychological parameters we developed a network of mutual relations by causal modelling using a modified method of path analysis following a similar reasoning as current dynamic causal modelling approaches used in imaging data [36], [37]. Briefly, crosstables of bivariate correlations were calculated within functional blocks of parameters (e.g. motor parameters). Subsequently, multiple correlations using the stepwise forward method of building regression equations was calculated to analyse which parameters were the dominant partners determining correlations between functional blocks of parameters. When the dominating parameter(s) had been identified (in some cases more than there one parameter represented a block), median split analysis was used to assign a direction of prediction (bidirectional, when median split groups predicted a significant difference in the correlated parameter, and vice versa; unidirectional, when median split groups predicted significant differences in the correlated parameter, but not vice versa). For description of this complex network of interrelations we used the wording “correlated” or “predicted” (implying causal relationship) to signify correlations that operated either bi- or unidirectional.

## Results

A total of 118 patients (91 female and 27 male; i.e. 77.1 and 22.9%) were enrolled in the study (Figure 1). Patients had a mean age of 58±12 years (range: 20–84 years. Mean duration of disease since inciting event was 42±23 months (range: 12–163 months; median 37.5 months). 105 patients were right-handed (89%), six patients were left-handed (5.1%), and seven patients were functionally bimanual (5.9%). The disease affected the right hand in 62 (52.5%) and the left hand in 56 patients (47.5%).

### Clinical Assessment

The average limb volume of the contralateral hand was 480.4±81.5 ml, while the volume of the affected hand was significantly reduced (469.2±83.4 ml; p<0.05; p<0.001 after correction for hand dominance. Volume reduction at the affected hand was pathological (reduction >5%, i.e. 24 ml) in 36/118 patients (31.3%), while only 16/118 (13.9%) exhibited pathological volume enlargement suggestive of hand edema. The quantitative assessments were corroborated by standardised clinical examination identifying 30/118 patients (25.4%) displaying clinically relevant signs of edema (assessment of volume reduction was not feasible by clinical examination).

Patients with chronic CRPS displayed a statistically significantly lower hand temperature at the affected hand albeit differences were small (hand dorsal affected: 32.7±1.7°C; contralateral: 33.0±1.5°C; p<0.01; hand palm: affected: 33.7±1.5°C; contralateral: 33.9±1.2°C; p<0.05). A total of 48 patients (40.7%) displayed abnormal skin colour at the affected hand, with 15 patients exhibiting glossy skin (12.7%). Twelve of these 15 patients displayed combined skin discolouration and glossy skin.

**Figure 2:**
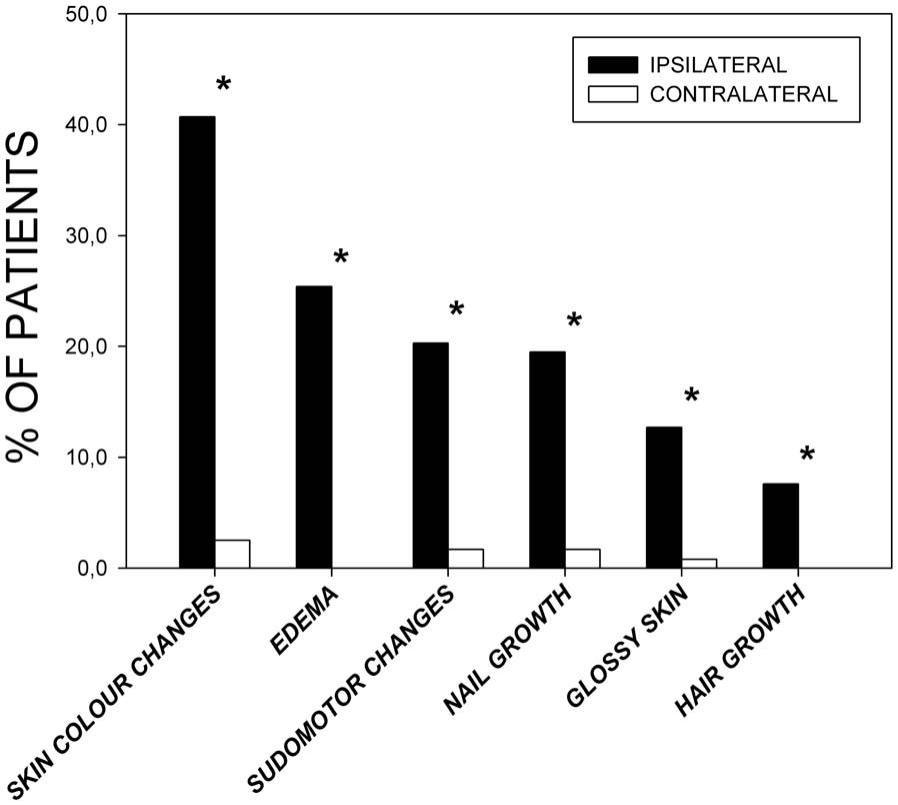
Clinical assessment of sudomotor dysfunction, throphic dysfunction, and edema. Bars represent the percentage of patients displaying the respective signs or symptoms. Significance: * p<0.05 ipsilateral hand vs. contralateral hand, Wilcoxon signed ranks method test.

Clinical assessment of sudomotor or trophic changes revealed the presence of enhanced sweating at the affected hand in 24 patients (20.3%), and in two patients (1.7%) at the contralateral hand. One of the two patients was affected bilaterally. Nine patients (7.6%) complained of augmented hair growth at the site of chronic CRPS, while disturbed nail growth was found in 23/118 patients (19.5%). Two patients had disturbed contralateral nail growth (1.7%), in one patient affecting solely the contralateral extremity (Figure 2).

**Figure 3:**
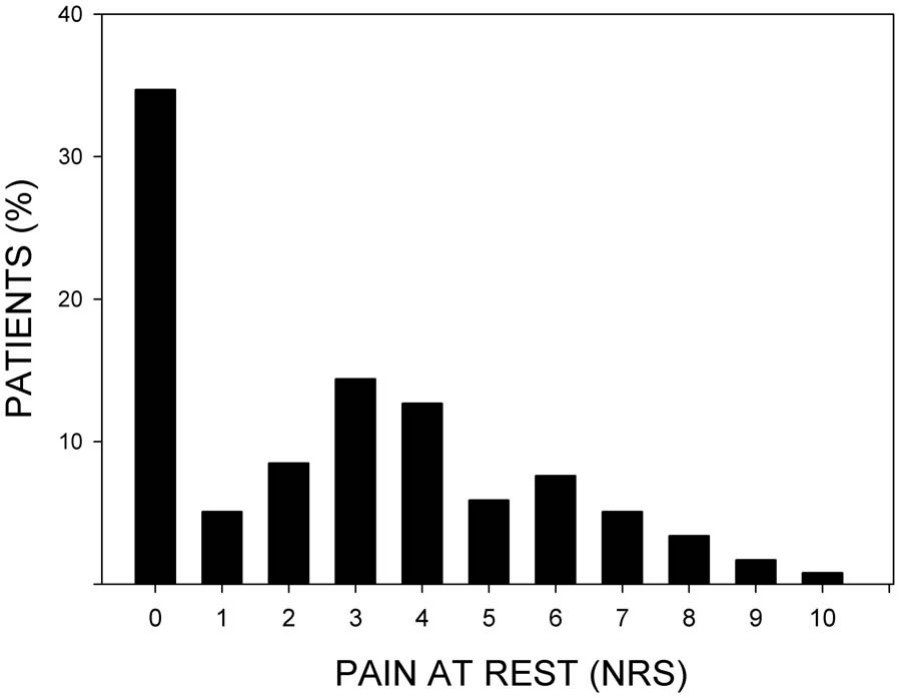
Pain at rest by means of Numeric Rating Scale (NRS). 0 indicates no pain at rest, and 10 indicating the worst pain. Bars display the percentage of patients in the respective category. The majority of patients (77/118 = 65.3%) reported spontaneous ongoing pain (44 patients with pain scores ≥4 = 37.3%). Mean NRS-score: 2.8±2.7; median NRS-score: 3.

The majority of patients (77/118 = 65.3%) reported spontaneous ongoing pain (44 patients with pain scores ≥4 = 37.3%). Only 41/118 patients were completely pain-free at rest (mean NRS-score: 2.8±2.7; median NRS-score: 3) (Figure 3). A few patients (4/118 = 3.4%) reported pain at rest at the contralateral hand (NRS scores ≥5 respectively). A substantial proportion of patients complained of exaggerated pain during physical strain of the hand (95/118 = 80.5% at the affected, and 13/118 = 11.0% at the contralateral hand). Furthermore, nearly half of the patients complained of secondary pain radiating to the ipsilateral elbow (57/118 = 48.3%), or shoulder (47/118 = 39.8%). On the contrary, the percentage of patients with pain at the contralateral elbow or shoulder was marginal. Interestingly, by means of clinical neurological examination using side to side differences, merely 11% of the patients were identified to show signs of hypoaesthesia, and 29.7% were classified as patients with clinical hyperalgesia (Table 2).

**Table 2:** Pain assessment and clinical somatosensory testing.

	Ipsilateral Hand	Contralateral Hand	
**Pain During Physical Strain**	80.5% (n = 95)	11% (n = 13)	
**Elbow Pain**	48.3% (n = 57)	6% (n = 7)	
**Shoulder Pain**	39.8% (n = 47)	5.1% (n = 6)	
**Mechanical Hyperalgesia**	29.7% (n = 35)	1.7% (n = 2)	P<0.001
**Hypoaesthesia**	11% (n = 13)	0.8% (n = 1)	P<0.001
**Mechanic Dynamic Allodynia**	5.1% (n = 6)	0%	P<0.05

### Quantitative Sensory Testing (QST)

#### Detection Thresholds

**Figure 4:**
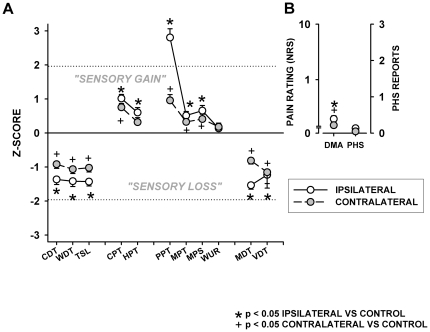
Standardized comparison of QST data normalized to mean and standard deviation of the control group (z-normalisation). A: Somatosensory profile of thermal and mechanical thresholds: Thermal Detection Thresholds: CDT: Cold Detection Threshold; WDT: Warm Detection Threshold; TSL: Thermal Sensory Limen. Thermal Pain Thresholds: CPT: Cold Pain Threshold; HPT: Heat Pain Threshold. Mechanical Pain Thresholds: PPT: Pressure Pain Threshold; MPT: Mechanical Pain Threshold; MPS: Mechanical Pain Sensitivity; WUR: Wind-up ratio. Mechanical Detection Thresholds: MDT: Mechanical Detection Threshold; VDT: Vibration Detection Threshold. B: PHS: Paradoxical Heat Sensation (PHS); Dynamic Mechanical Allodynia (DMA). Significance: ipsilateral hand vs. control: * p<0.05. Significance contralateral hand vs. control: + p<0.05. Patients with chronic CRPS displayed a bilateral hyperalgesia in every painful somatosensory modality as well as bilateral somatosensory loss.

Patients exhibited a highly significant loss of thermal (z-values for CDT: −1.37±1.57; WDT: −1.42±1.66; and TSL: −1.43±1.37) and mechanical detection (MDT: −1.53±1.29; VDT: −1.15±1.97) at the affected hand compared to age and gender-matched healthy controls (all p<<0.0001). Notably, the same highly significant thermal as well as mechanical hypoesthesia also occurred at the contralateral hand, albeit to a lesser extent (CDT: −0.92±1.31; WDT: −1.07±1.36; TSL: −1.02±1.32; MDT: −0.81±1.07; VDT: −1.23±2.30; all p<<0.0001). Thus, sensory loss was homogeneously encountered in any non-painful somatosensory modality. Average somatosensory loss was −1.38 standard deviations (z-values) at the affected hand, and −1.01 SD at the contralateral hand (Figure 4).

#### Pain Thresholds

Thresholds for cold and heat pain were significantly decreased, indicating cold and heat hyperalgesia (HPT: 0.60±1.50; CPT: 1.01±1.23; both p<0.001). Additionally, a striking hyperalgesia to painful blunt pressure was found (PPT: 2.81±2.75; p<<0.0001). Likewise, the pain threshold to pin prick was lowered, and pain ratings to suprathreshold pin pricks were increased (MPT: 0.51±1.24; MPS: 0.66±1.66; both p<0.001). Moreover, a statistically significant, albeit small degree of dynamic mechanical allodynia occurred (DMA: 0.19; log10 pain rating −0.710±0.639; p<0.01). However, pain summation at the affected hand remained unaltered (WUR: 0.14±0.93; p = 0.27). Significant hyperalgesia was also encountered contralaterally throughout all pain parameters (CPT: 0.75±1.22, p<0.0001; HPT: 0.32±1.40, p = 0.052 PPT: 0.96±1.84, p<0.0001; MPT: 0.33±0.87, p<0.01; MPS: 0.41±1.53, p<0.05; DMA: 0.15; log10 pain rating −0.822±0.475; p<0.01). Although marginally more frequent, the incidence of paradoxical heat sensations (PHS) was indifferent from healthy controls (0.14±0.50 ipsilateral, 0.11±0.57 contralateral; p = 0.18 and 0.30, respectively). Thus, sensory gain, i.e. hyperalgesia was encountered in any painful somatosensory modality. Average pain sensitivity was above normal thresholds by +1.12 standard deviations at the affected hand, and +0.55 standard deviations at the contralateral hand. QST results are summarized in Figure 4.

### Assessment of Hand Motor Function

**Figure 5:**
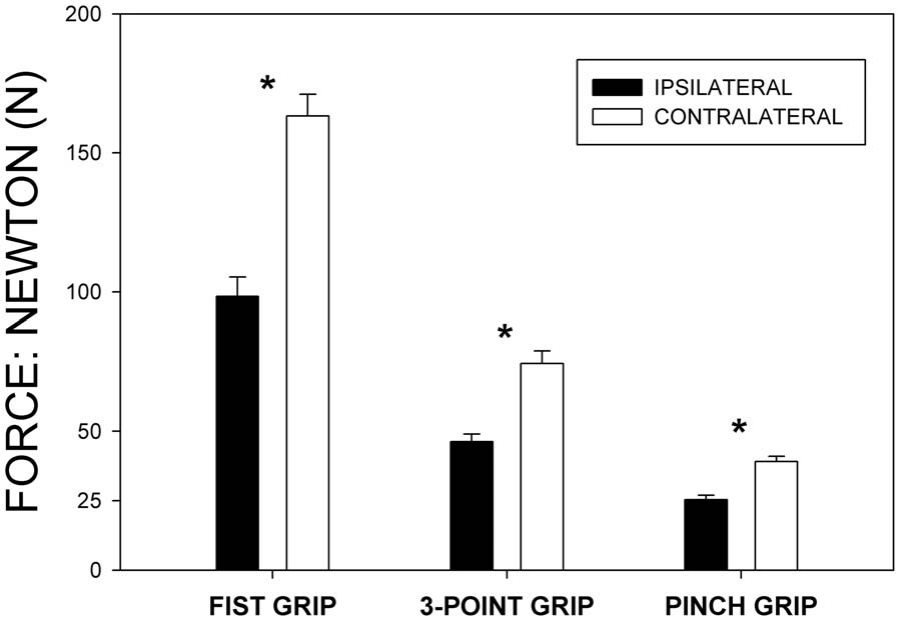
Hand force in chronic CRPS. Bars show a homogeneous reduction of hand force in Newton as compared to the contralateral side throughout the applied tasks. Significance: * p<0.001 ipsilateral vs. contralateral hand, paired t-test.

Hand force at the affected hand as measured by dynamometry was significantly reduced in any test. Force reduction (−35–40%) was homogeneous throughout all tests performed, namely 98.4±74.8 N vs. 163.4±83.38 N for fist grip, 46.2±30.0 N vs. 74.4±48.4 N for three point grip, and 25.4±17.6 vs. 39.1±19.9 N for pinch grip; affected vs. contralateral hand respectively; all p<0.001 (Figure 5).

**Figure 6:**
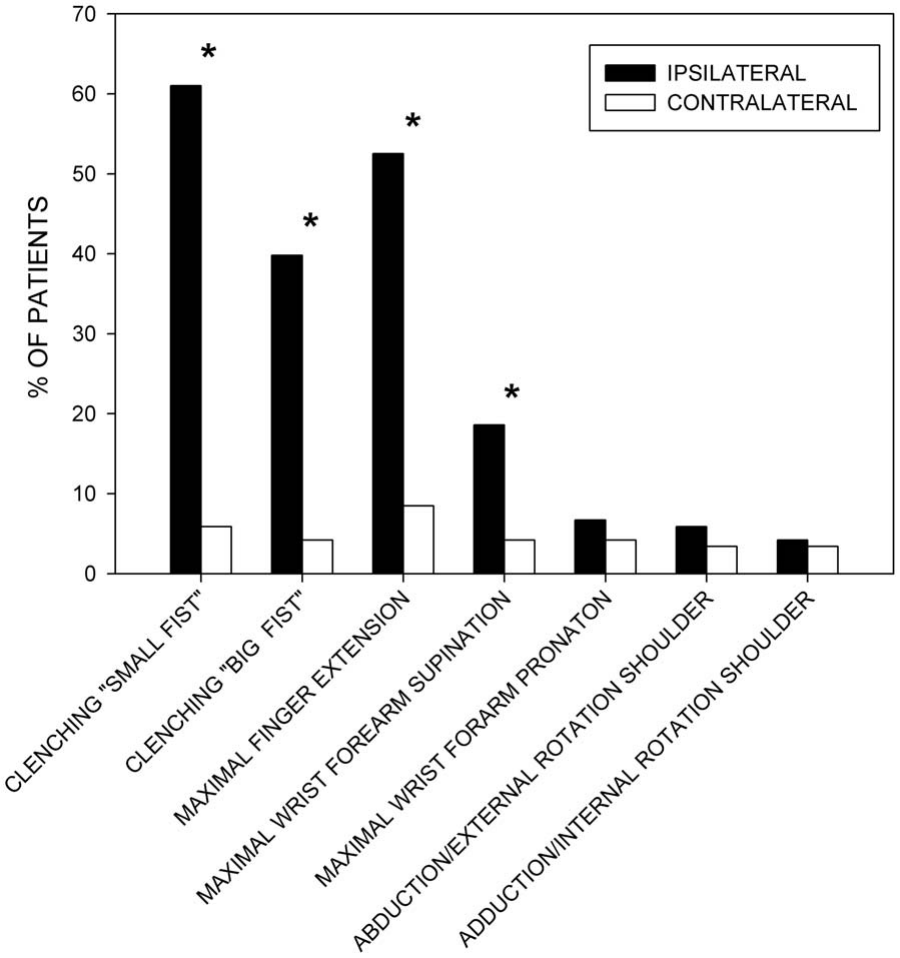
Impairment of hand motor function. Bars display the percentage of patients displaying the respective motor impairment. Significance: * p<0.001 ipsilateral vs. contralateral hand, Wilcoxon signed ranks method test.

Patients with chronic CPRS displayed significant motor impairments as revealed by several measures, including the ability to clench a fist, to maximally extend the fingers, or to supinate the wrist and forearm (all p<0.001 compared to the contralateral side). However, impaired pronation of the wrist or forearm, as well as a disability to rotate the ipsilateral shoulder was rarely seen (Figure 6). Furthermore, active range of wrist motion (flexion and extension), thumb abduction as well as maximally achievable hand extension were all considerably reduced (all p<0.001 compared to the contralateral side). The majority of patients (71/118 = 60.2%) were unable to completely clench a fist at the affected hand (only 7/118 = 5.9% at the contralateral side). Mean deflection deficit (all patients) at the affected hand was 23.7±24.5 mm, and 40.1±19.7 mm in patients with deflection deficits. Moreover, 19 patients displayed a D1–D5 opposition deficit at the affected hand (all patients 6.5±19.3 mm; 39.2±31.6 mm in the 19 patients with opposition deficit). Contralateral D1–D5 finger opposition deficit was only seen in 2 patients (40 mm and 50 mm, respectively). Table 3 summarises these motor results.

**Table 3:** Active range of motion.

	Ipsilateral Hand	Contralateral Hand	p-Value
**Wrist Extension**	52.2°±14.5°	63.5±11.5°	<0.001^a^
**Wrist Flexion**	56.3°±16.5°	70.2±10.5°	<0.001^a^
**Thumb Abduction**	50.8±9.7°	55.±8.7°	<0.001^a^
**Diameter D1–D5**	16.8±3.7 cm	19.2±2 cm	<0.001^a^
**Deflection Deficit**	2.4±2.4 cm	0.3±1.2 cm	<0.001^a^
**Opposition Deficit D1–D5**	0.6±1.9 cm	0.1±0.56 cm	<0.01^a^

a Paired t-test.

Total SODA score amounted to 88.4±22.8, and average SODA pain-score was 2.1±3.2 (SODA pain-score ranges from 0 to 12). The overall score of the Michigan Hand Outcome Questionnaire (MHQ) was 53.0±15.0 (range: 22–84). Furthermore, all subscores of the MHQ displayed significant hand disability (Table 4).

**Table 4:** Michigan Health Questionnaire: (MHQ).

	*Score*
Overall Hand Function (MHQ I)	48.75°±°24.1
Activities of Daily Lifer (MHQ II)	44.75°±°30.3
Work Performance (MHQ III)	50.5°±°25.3
Pain (MHQ IV)	47.6°±°28.9
Aesthetics (MHQ V)	65.5°±°25.57
Satisfaction with Hand Function (MHQ VI)	56.9°±°23
**Overall MHQ Score**	**53**°**±**°**15**

Hand disability measured with MHQ: For the pain scale, higher scores indicate more pain. For the other scales, higher scores indicate better hand performance. Scores are normalized to a range from 0–100.

### Psychological Assessment and Health-Related Quality of Life

Pain disability index averaged 24.2±20.3. Average CES-D scores were 37.0±11.3, thereby considerably exceeding the limit (CES-D: >27) to diagnose a depressive episode in pain patients. Notably, 97/118 patients (82%) had a CES-D score of above 27. Mean PTSS-10 sum score amounted to 28.7±12.7 (median score: 27). 32/118 patients (27.4%) displayed a PTSS-10 higher than the cut-off of 35 reported in the literature. The majority of patients with chronic CRPS (89/118 = 75.4%) reported multiple traumatic memories (Table 5). Mean number of traumatic memories was 1.48±1.24. Patients with chronic CRPS displayed significantly decreased health related quality of life in seven of the eight dimensions of the SF36 (p<0.0015), with the only exception of the dimension vitality (Table 6).

**Table 5:** Number of traumatic memories.

	%	(n)	PTSS-10 Score
**No Traumatic Memories**	24.6	17	17.3°±°7.2
**One Traumatic Memory**	33.3	23	21.9°±°6
**Two Traumatic Memories**	20.3	14	36.5°±°10.8
**Three Traumatic Memories**	13	9	37.9°±°11.2
**Four Traumatic Memories**	8.7	6	45.4°±°12.7
**Mean Number of Traumatic Memories**	1.48°±°1.244		28.7°±°12.7

**Table 6:** Health related quality of life: (SF-36).

	Mean±SD	Z-Score	p
PF	58.2°±°26.6	−1.1°±°1.2	<0.05
RP	45.8°±°39.3	−1°±°1.2	<0.05
BP	57.3°±°23	−0.8°±°1	<0.05
GH	47.7°±°11.4	−1.2°±°0.6	<0.05
VT	58.4°±°12.8	−0.1°±°0.6	n.s.
SF	70.6°±°13.4	−0.6°±°0.6	<0.05
RE	52.4°±°40.8	−0.9°±°1.2	<0.05
MH	49°±°14.8	−1.4°±°0.8	<0.05
PCS	41.5°±°10	-	
MCS	41.9°±°4.6	-	

The scales of the SF-36 score from 0–100, with 0 indicating worst health and 100 the best.

PF: Physical Functioning.

RP: Role Limitations, Physical:

BP: Bodily Pain.

GH: General Health.

VT: Vitality.

SF: Social Functioning.

RE: Role Limitations, Emotional.

MH: Emotional Well-Being.

PCS: Physical Component Summary Score MCS: Mental Component Summary Score.

t0: Before beginning of treatment.

t1: One year after beginning of treatment.

n.s.: Not significant.

Z-Score: SF-36 data were normalized to a US-General population (n = 2393).

### Differences between Patients with “Warm” and “Cold” CRPS and Patients Showing Significant Hand Discoloration

Merely three patients displayed a temperature difference of more than +1°Celsius and were therefore classified as warm CRPS, while sixteen patients had a temperature difference between the ipsi- and the contralateral hand of ≥−1°C and were therefore classified as “cold” CRPS. Patients with “warm” CRPS displayed more spontaneous pain, (NRS: 5.7±2.1; PDI 51±12.8) than patients with “cold” CPRS (NRS: 3.3±2.8; PDI: 30.6±22); but differences were not statistically significance probably due to the small number of patients in each group. Furthermore, no differences in hand volumes or significant differences in QST between patients with “warm” or cold CRPS could be detected. Contrariwise, patients displaying hand discoloration at the time of assessment, had significantly more spontaneous pain (NRS: 4±2.5; PDI 32±19.1) compared to patients without signs of hand discoloration (NRS: 1.9±2.4; PDI 18.8±19.6) (p<0.001). Furthermore, patients with hand discoloration displayed significantly more pressure pain hyperalgesia (Z-values for PPT: 2.01±1.97 in patients without hand discoloration; PPT: 3.96±3.33 in patients displaying hand discoloration; p<0.001). Moreover, no other ipsi- or contralateral changes in QST parameters or significant differences in hand volume could be detected.

### Interdependence of Pain, Stress, Depression, Sensory Changes and Hand Motor Function

**Figure 7:**
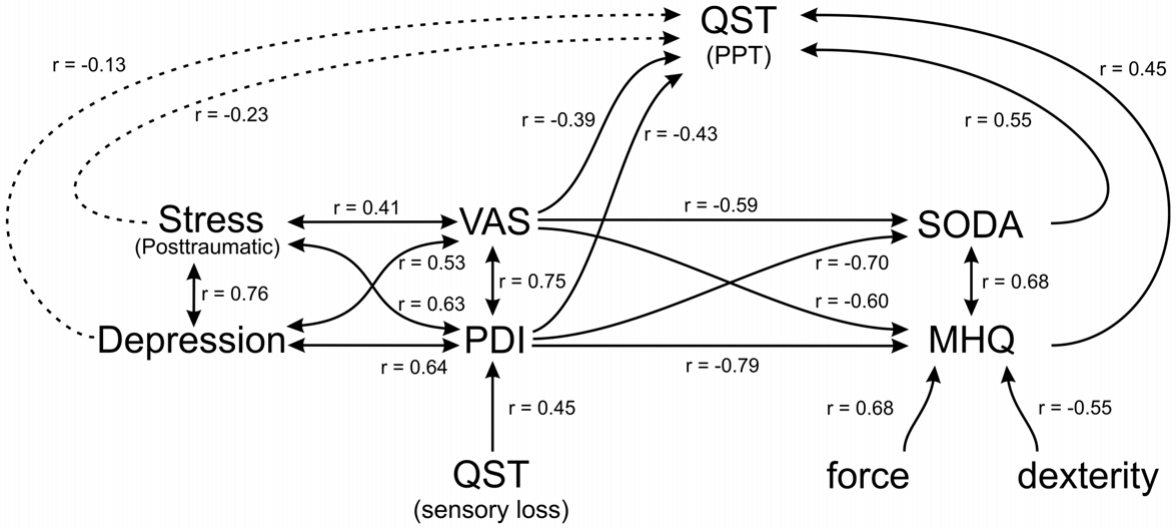
Overview on correlational and predictive interaction of sensory function, pain, hyperalgesia, and motor and psychological functioning. Multiple correlation was calculated to analyse correlations between functional blocks of parameters using the stepwise forward method of building regression equations. Median split analysis was then applied to assign direction of influence of parameters included in the regression equation, i.e. to identify predictors.

Correlation analysis encompassing all estimated parameters revealed several functional complexes. Ongoing pain and pain-related disability (PDI) (r = 0.75, p<0.0001), as well as posttraumatic stress (PTSS-10) and depression (CES-D) were highly correlated (r = 0.76, p<0.0001). There were also highly significant correlations between these two blocks of parameters (r = 0.41–0.64, all p<0.0001). Median split analysis suggested that none of these relationships was unidirectional (Figure 7). Additionally, pain and PDI were significantly predicted by sensory loss (combined thermal and mechanoreceptive deficit), which remained highly significant in multiple correlation analysis even after removing the stronger impact of stress and depression (partial r = 0.37 and 0.45, both p<0.0001). Median split analysis revealed that pain and pain-related disability were significantly predicted by the magnitude of sensory loss, but not vice versa.

Scores of both methods of comprehensive objective and subjective motor function assessment (SODA and MHQ) were strongly predicted by ongoing pain (r = −0.59 and r = −0.60, both p<0.0001) and pain-related disability (r = −0.70 and r = −0.79, both p<0.0001). Median split analysis revealed that VAS or PDI were highly significantly predictive for differences in SODA or MHQ (all p<0.0001), but not vice versa (all p>0.20) suggesting that motor disturbances were predicted by pain and PDI rather than correlated.

Hyperalgesia was analysed using the QST parameter exhibiting the most prominent gain, namely blunt pressure pain (PPT). Combined correlation and median split analysis revealed that hyperalgesia to blunt pressure stimuli was highly significantly predicted by motor dysfunction (SODA and MHQ, both p<0.0001), and to a lesser albeit significant degree by pain or PDI (both p<0.0001). In contrast, hyperalgesia was only weakly predicted by posttraumatic stress (r = −0.23; p<0.05) and not at all by depression (r = −0.13, p = 0.17). An overview on correlational and predictive interaction of sensory function, pain, hyperalgesia, and motor and psychological functioning is depicted in Figure 7.

## Discussion

Chronic CRPS was characterized by chronic pain at rest and during exercise, combined with substantially limited hand force and impaired motor function. Accordingly, the resulting overall hand function was substantially disabled even several years after the initial trauma. Chronic CRPS patients exhibited pronounced bilateral sensory changes revealed by QST, namely hyperalgesia and sensory loss. Furthermore, patients exhibited a clinically relevant degree of chronic stress as well as signs of depression. In contrast, patients with chronic CRPS displayed only marginal vasomotor, trophic or sudomotor changes.

### Pain and somatosensory changes in chronic CRPS

The majority of patients with chronic CRPS complained about spontaneous pain. The observed proportion is comparable to outcome studies in patients with childhood-onset of CRPS and a median follow up after 12 years, [38]. However, a comprehensive population based study by de Mos et al. examining chronic CRPS in adults reported spontaneous pain in about 30% of all cases [39]. The large number of patients complaining of pain during physical strain as well as relevant pain-related interference with activities of daily life, indicated by the elevated PDI, further illustrates the impact of pain on patients even several years after the initial trauma. The somatosensory QST profiles display a combination of a bilateral mechanical and thermal hyperalgesia, with the hallmark sign of blunt pressure hyperalgesia, as well as bilateral thermal and mechanical hypoaesthesia of a similar magnitude in all thermal and mechanical non-nociceptive modalities. Sensitivity was uniformly lowered by approximately 1.4 standard deviations ipsilaterally and 1.0 standard deviations contralaterally, comparable to previous findings [13], [14], [40]. Elevated detection thresholds for mechanical and thermal stimuli can be attributed to a loss of small Aδ and C-fibers, thereby supporting the potential role of small fiber loss in the pathophysiology of the disease [5]. However, in the absence of structural changes, tactile hypoaesthesia might be generated by functional impairments of sensory pathways, namely a central inhibition of non-noxious pathways [14]. Heterosynaptic long term potentiation (LTP) of inhibitory spinal interneurons has been discussed as a possible pathomechanism in this context [41]. The combination of a distinct cold hyperalgesia, absence of PHS and a minor heat pain hyperalgesia, likewise indicates a preponderance of small fibre degeneration, while QST signs of inflammatory hyperalgesia were almost absent [13], [42]. The relative contribution of Aδ- and C-nociceptors to pressure pain threshold is only partially resolved and peripheral as well as central mechanisms of action are supposed to be involved [19], [43], [44]. The bilateral somatosensory changes, which have been described for thermal thresholds before, likewise suggest the involvement of spinal or supraspinal structures [13]. Therefore, in chronic CRPS signs of small fibre degeneration combined with indicators of a central sensitization dominate the QST results. Importantly, the clinical somatosensory examination was not sufficiently capable to reveal these pronounced somatosensory changes, stressing the importance and higher sensitivity of a standardized QST examination.

### Motor Dysfunction in Chronic CRPS

Because motor dysfunction was not a “conditio sine qua non” in former diagnostic criteria of CRPS, many studies do not report the amount of motor changes in CRPS or confine to describe manifestations of existing motor deficits [1], [45], [46]. Hand force was substantially lowered by about 40% throughout all tests conducted, thereby exceeding the previously reported amount of force impairment in patients with chronic CRPS [24]. The differences in active range of motion found are conspicuous, when the affected and unaffected hands are compared. However, the results were still within a range required to perform activities of daily life [24]. The deficits in finger movements, as quantified by the impaired fist clenching ability and deflection/opposition deficits have not been quantified before. The pathophysiology of ongoing motor dysfunction in this late stage of the disease might be attributed to conglutinations of peripheral joints and tissues as signalled by the deficits in finger and wrist movements, thereby representing long-term consequences of an initial inflammatory process. However, these impairments can likewise be attributed to adaptive changes in central motor processing characterized by lower levels of motor cortical activation [9], [47]. Moreover, ongoing chronic pain accounting for increasing fear of movement, might similarly contribute to the measured level of hand function impairment. Kinesophobia as well as the level of ongoing pain have both been associated with a decreased level of muscle activation in a model of chronic musculoskeletal neck pain [48]. A proper distinction between peripheral or central motor changes or the degree of kinesophobia was impossible, as all movement tasks were only actively performed by the patients.

### Vasomotor-, sudomotor-, trophic dysfunction and edema in chronic CRPS

In contrast to the distinct somatosensory and motor alterations, other symptoms of CRPS were by far less present. Of these changes, skin discolouration was most frequently noted with an incidence of about 40%. In contrast to patients with acute CRPS, in which hand edema and clinical aspects of a “warm” hand dominates, the volume of the affected hand was significantly reduced when compared to the contralateral side, and on average the hand affected by CRPS appeared “colder” [49]. Both, edema as well as increased skin temperature, signal ongoing peripheral inflammation [50]. Moreover, the clinical signs of autonomic dysfunction like hyperhidrosis and alterations of hair growth were only moderately present, when compared to acute CRPS [45]. As the presence of an inducible autonomic nervous system autoantigen has been recently demonstrated in CRPS patients, one may speculate about possible interactions between autoimmune and inflammatory processes dominating the acute phase of CRPS, whereas in chronic CRPS the clinical signs of peripheral neurogenic inflammation and autonomic dysfunction may have mostly subsided due to post-inflammatory burn-out.

Recently, it has been suggested that patients with “warm” and “cold” CRPS (i.e. a significantly warmer or colder hand affected by CRPS when compared to the contralateral side), might be representatives of a more peripheral or central pathophysiology of the disease [14]. Furthermore, it has been demonstrated that patients initially classified as “cold” CRPS showed more pain when re-evaluated several years after the initial assessment [3]. “Warm” CRPS seems to be predominant in the acute phase of CRPS, where signs of peripheral neurogenic inflammation dominate [13]. Therefore, as patients in our study suffered from chronic CRPS with duration of disease of at least one year, only few patients with “warm” CRPS could be identified, and no differences between “warm” and “cold” CRPS could be detected. In addition, we were unaware of the patient's initial classification into either “warm” or “cold” at the time the CRPS was diagnosed.

While only few patients displayed signs of significant temperature differences between both hands, a considerable proportion of patients with chronic CRPS showed hand discoloration, which can, like temperature differences, also be interpreted as a sign of an ongoing vasomotor instability [51]. These patients had significantly more spontaneous pain, when compared to those without apparent skin discoloration. While the pathophysiological meaning of skin discoloration remains unclear, it might nevertheless serve as an easy to detect clinical sign in order to evaluate the severity of the ongoing disease.

### Stress and Depression in Chronic CRPS

While psychological factors do not seem to be associated with the onset of CRPS [52], patients in this study showed clinically relevant levels of both chronic stress and depression. Their median PTSS-10 score was comparable to patients surviving life threatening events resulting in long-term critical care therapy like sepsis or acute respiratory distress syndrome (ARDS) [53]. Recently, a diffusion tensor imaging study of patients with CRPS displayed grey and white matter abnormalities in the ventromedial prefrontal cortex, a brain area associated with the development and maintenance of posttraumatic stress disorder (PTSD) [54], [55]. Furthermore, CRPS patients exhibit signs of an impaired innate immunity, presumably reflecting the immunological consequences of an immunosuppressive neuroendocrine stress response [32]. Interestingly, CRPS has been reported to be elicited by stress exacerbation in patients with PTSD [56]. Furthermore, PTSD patients show high rates of concomitant chronic pain, and the severity of chronic pain in PTSD seems to be closely correlated to the degree of stress [56]. Likewise, substantial correlations were shown in this study between the levels of stress and depression, and the degree of ongoing pain and pain-related disability. Finally, concerning somatosensory thresholds, in analogy to the correlation between evoked pain (PPT) and stress levels in this study, an influence of PTSD and depressive symptoms on QST results has been described [11], [12]. Summarized, similar to critical care patients suffering from sepsis, onset of PTSD in patients with CPRS might be triggered by an inciting inflammatory event, but in contrast to critical care patients in CRPS, chronic stress may perpetuate the progression of the disease [57]. This hypothesis is further supported by combined correlation and median split analysis, showing a significant prediction of hyperalgesia and motor dysfunction by the closely interrelated cluster of ongoing pain, pain-related disability, stress and depression (Figure 7).

### Hand Disability, Dexterity and Central Maladaptation in Chronic CRPS

Chronic CRPS patients displayed a considerable degree of hand disability and impaired hand dexterity, comparable to patients with rheumatoid arthritis and mobile swan neck deformities [58]. Hence patients with chronic CRPS sustain a pronounced interference with the accomplishments of daily life, even years after the initial trauma, further emphasized by the reduced general health related quality of life. This is emphasized by the high rate patients found to be incapable to work even several years after the initial treatment in a study by de Mos et al [39]. The dissection of factors possibly influencing the degree of hand disability revealed that the degree of ongoing pain and pain-related disability was the most important factor in prediction of manual motor disturbance (see Figure 7). This suggests a participation of higher central mechanisms of (mal-)adaptation.

Chronic pain disrupts the so-called “default mode network” of the brain [59] and may lead to widespread structural changes of the in animal as well as human brain [60], [61] Reduced neuronal metabolism in several brain regions has been demonstrated by magnetic resonance spectroscopy [62]. These maladaptive plasticity mechanisms of the CNS are also known from pathologies like phantom limb-related disorders [63] Similar maladaptive plasticity also occurs in CRPS [64]–[66], but may be reversible by successful rehabilitation and concomitant pain reduction [65], [67] Several structural changes suggestive of functional loss have been demonstrated in the brain of CRPS patients, e.g. regional atrophy in brain areas involved in pain processing, like insula and prefrontal cortex, as well as decreased connectivity between prefrontal cortex and the basal ganglia [54]. The basal ganglia, especially the putamen, are not only involved in descending pain control but moreover in the pathophysiology of other motor disorders, like Parkinson's disease or restless legs syndrome [68]–[70]. These syndromes are also presenting with spontaneous pain and/or hyperalgesia by hitherto unexplained mechanisms [71], [72]. Recently, a net shift from inhibitory towards facilitatory pain control has been demonstrated in CRPS patients [73]. In aggregate, there is ample evidence on putative central mechanisms suggesting that the motor disorders in CRPS may be elicited by the level of concomitant pain. However, due to the study design, the results could not be compared to a similar group of patients suffering from other chronic pain syndromes like fibromyalgia or chronic low back pain. Therefore it remains unclear if these interactions between chronic pain, stress, depression and motor disorder are specific features of CRPS, or just reflect a general characteristic of any kind of chronic pain [74].

### Study Limitations

As the study design is not prospective, a potential recruitment bias must be taken into account. Overall, 86 patients which had been contacted, refused to participate in the study. Moreover, 67 eligible patients could not be contacted, and six patients deceased (Figure 1). However, only few of the patients refusing to participate in the study reported to be pain-free, while others reported that participating in the study would be too time consuming, and some indicated to be discontent with prior treatment at the pain clinic.

QST is a behavioural functional measure of somatosensory function, and none of the performed tests provides a direct evidence of structural changes in either the peripheral or the central nervous system [75], [76]. Furthermore, the applied therapies were not prospectively controlled, but followed the standard therapy guidelines of our pain department at the time of the patients' initial attendance. Therefore, possible interactions resulting from different therapeutic regimens can not be ruled out, but are unlikely due to the high number of patients included in the study.

**Figure 8:**
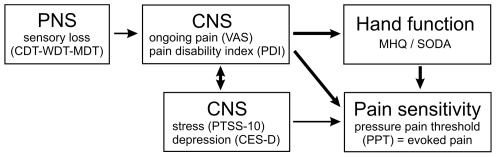
Suggested model of functional connection between sensory loss, the degree of ongoing “central” pain, pain sensitivity, depression and stress, and the degree of hand dysfunction in chronic CRPS. Based on multiple regression analysis the proposed model holds that initial sensory loss, possibly caused by a peripheral neurogenic inflammation, accounts for the induction of ongoing pain. The degree of ongoing “central” pain, depression and stress, determines the development and degree of hand dysfunction and the amount of evoked pain in chronic CRPS. The impact of parameters is depicted by the width of arrows, representing the respective correlation coefficients.

Taken together, one can speculate about the following mechanisms interacting in the pathophysiology of CRPS: Initial sensory loss, possibly caused by a peripheral neurogenic inflammation, accounts for the inducting of ongoing pain. The degree of ongoing “central” pain, depression and stress, determines the development and degree of hand dysfunction and the amount of evoked pain in chronic CRPS (Figure 8). In summary, chronic CRPS in characterized by a combination of chronic ongoing as well as evoked pain, a distinct level of stress and depression, resulting in a disabled hand function even years after the impressive symptoms of acute CRPS like edema and sympathetic dysfunction subside. Hence, in the future enhanced efforts should be made to set up tailored treatment strategies targeting underlying pathomechanisms in order to improve long term outcome even in severe cases of CRPS.
